# The plant-like protein phosphatase PPKL regulates parasite replication and morphology in *Toxoplasma gondii*

**DOI:** 10.1186/s13071-024-06135-6

**Published:** 2024-03-18

**Authors:** Xi-Ting Wu, Xu-Wen Gao, Qiang-Qiang Wang, Kai He, Muhammad Saqib Bilal, Hui Dong, Yi-Dan Tang, Hui-Yong Ding, Yue-Bao Li, Xiao-Yan Tang, Shaojun Long

**Affiliations:** 1 National Key Laboratory of Veterinary Public Health and Safety, and School of Veterinary Medicine, China Agricultural University, Beijing, 100193 China; 2 The Key Laboratory of Plant Immunity, Nanjing Agricultural University, Nanjing, 210095 China

**Keywords:** *Toxoplasma gondii*, Plant-like phosphatase, PPKL, Cytoskeleton, Parasite replication, Parasite morphology

## Abstract

**Background:**

The protozoan parasite *Toxoplasma gondii* encodes dozens of phosphatases, among which a plant-like phosphatase absent from mammalian genomes named PPKL, which is involved in regulating brassinosteroid signaling in *Arabidopsis*, was identified in the genome. Among the Apicomplexa parasites, *T. gondii* is an important and representative pathogen in humans and animals. PPKL was previously identified to modulate the apical integrity and morphology of the ookinetes and parasite motility and transmission in another important parasite, *Plasmodium falciparum*. However, the exact function of PPKL in the asexual stages of *T. gondii* remains unknown.

**Methods:**

The plant auxin-inducible degron (AID) system was applied to dissect the phenotypes of PPKL in *T. gondii*. We first analyzed the phenotypes of the AID parasites at an induction time of 24 h, by staining of different organelles using their corresponding markers. These analyses were further conducted for the parasites grown in auxin for 6 and 12 h using a quantitative approach and for the type II strain ME49 of AID parasites. To further understand the phenotypes, the potential protein interactions were analyzed using a proximity biotin labeling approach. The essential role of PPKL in parasite replication was revealed.

**Results:**

PPKL is localized in the apical region and nucleus and partially distributed in the cytoplasm of the parasite. The phenotyping of PPKL showed its essentiality for parasite replication and morphology. Further dissections demonstrate that PPKL is required for the maturation of daughter parasites in the mother cells, resulting in multiple nuclei in a single parasite. The phenotype of the daughter parasites and parasite morphology were observed in another type of *T. gondii* strain ME49. The substantial defect in parasite replication and morphology could be rescued by genetic complementation, thus supporting its essential function for PPKL in the formation of parasites. The protein interaction analysis showed the potential interaction of PPKL with diverse proteins, thus explaining the importance of PPKL in the parasite.

**Conclusions:**

PPKL plays an important role in the formation of daughter parasites, revealing its subtle involvement in the proper maturation of the daughter parasites during division. Our detailed analysis also demonstrated that depletion of PPKL resulted in elongated tubulin fibers in the parasites. The important roles in the parasites are potentially attributed to the protein interaction mediated by kelch domains on the protein. Taken together, these findings contribute to our understanding of a key phosphatase involved in parasite replication, suggesting the potential of this phosphatase as a pharmaceutic target.

**Graphical Abstract:**

**Figure:**
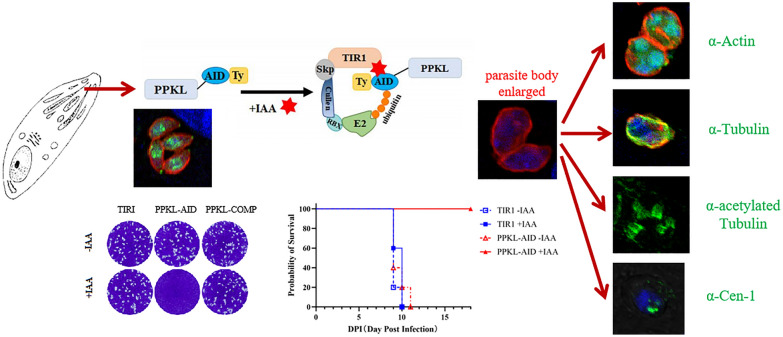

**Supplementary Information:**

The online version contains supplementary material available at 10.1186/s13071-024-06135-6.

## Background

*Toxoplasma gondii* is an obligate intracellular protist that belongs to the phylum Apicomplexa of the Alveolata. Apicomplexan parasites have a unique structure called the apical complex, which is highly conserved and completely specific among the more than 5000 species of Apicomplexa. In addition to *Toxoplasma*, Apicomplexan protozoa include *Plasmodium*, *Cryptosporidium*, and *Babesia*, which are important pathogens of humans and animals. Among them, *T. gondii* causes toxoplasmosis, an important disease worldwide and one of the most common foodborne infections [1]. *Toxoplasma gondii* can infect almost all homeotherms and proliferate in all nucleated cells, leading to host cell lysis [2]. Human toxoplasmosis infection generally does not show obvious clinical symptoms. Toxoplasmosis can be caused when the host immunity is low or defective, and it can lead to abortion or fatal injury to the fetus [3]. Nowadays, the potential impact of toxoplasmosis may exceed traditional recognition. Likewise, it is critical to identify and characterize unique factors that play key roles in *Toxoplasma* biology and pathogenesis for future drug development.

In contrast to the extensive research data emphasizing the importance of parasite kinases, the functions of most parasite protein phosphatases have not been extensively explored. Protein phosphatases can usually be classified into three groups according to their target amino acids: serine/threonine phosphatases, tyrosine phosphatases, and dual-specificity phosphatases. Among them, phosphoprotein phosphatases (PPPs) belonging to the serine/threonine protein phosphatases can be further subdivided into PP1, PP2A, PP2B, PP4, PP5, PP6, and PP7.

In recent years, the role of protein phosphatases in *T. gondii* has received considerable attention. Protein phosphatases play key roles in the invasion, proliferation, egress, motility, and bradyzoite transformation of *T. gondii*. Deletion of PP1 results in reduced invasion and delayed lysis of host cells [4]. PP2A plays an essential role in bradyzoite differentiation and affects tachyzoite growth and virulence [5]. PP2B regulates the adhesion of tachyzoites to host cells [6]. PP7 deletion reduces the plaque formation ability and virulence of tachyzoites [7]. Metal-dependent protein phosphatase 5C (PPM5C) is a PP5C-class serine/threonine phosphatase. It is localized on the plasma membrane of *Toxoplasma* and plays a regulatory role in attachment to host cells [8]. In addition, protein phosphatases regulate the complex environment of the parasitophorous vacuole and influence the association between the parasites and host cells. For example, one PP2C has been shown to work with casein kinase II to regulate the phosphorylation state of toxoflin, an actin-binding protein known to be secreted into the host [9], and PPM3C has a role in affecting the transport of specific effector proteins from parasitophorous vacuoles to host cells [10]. Moreover, a highly conserved tyrosine phosphatase, PRL, is involved in regulating intracellular Mg^2+^ homeostasis, affecting the attachment of the parasites to host cells, and is an important regulator of the lytic cycle and virulence [11]. It has also been shown that TgLaforin contains a dual-specificity phosphatase domain, which lays the foundation for targeting this phosphatase to prevent bradyzoite reactivation and transmission [12].

Besides those phosphatase homologs from the seven subfamily members common to the eukaryotes, apicomplexan parasites have three other serine/threonine protein phosphatases that are absent in mammals. One of these is a protein phosphatase with a kelch-repeat domain called PPKL, which is only found in plants and alveolates. Its kelch domain usually has five to seven kelch-repeat sequences, forming a β-propeller tertiary structure usually involved in protein–protein interactions. Four PPKL members were identified in *Arabidopsis*: BSU1, BSL1, BSL2, and BSL3. PPKLs in *Arabidopsis* are involved in regulating brassinosteroid signaling [13]. Among Apicomplexa protozoa, PPKL was analyzed in the ookinetes of *Plasmodium*, which showed that PfPPKL modulates the apical integrity and morphology of the ookinetes and parasite motility and transmission [14]. However, the function of PPKL in *T. gondii* remains unknown.

This study shows that TgPPKL is partially localized to the apical complex and parasite cytoplasm in the tachyzoite of *T. gondii*. Reverse genetic analysis suggests that PPKL is critical for parasite growth, and its depletion leads to morphological abnormalities and loss of parasite growth in mice. Taken together, the results of this study highlight the function of TgPPKL in the maturation of daughter parasites and the parasite cytoskeleton integrity, demonstrating the potential of this protein phosphatase as a novel therapeutic target.

## Results

### Identification of TgPPKL and generation of parasite line for protein depletion

**Fig. 1:**
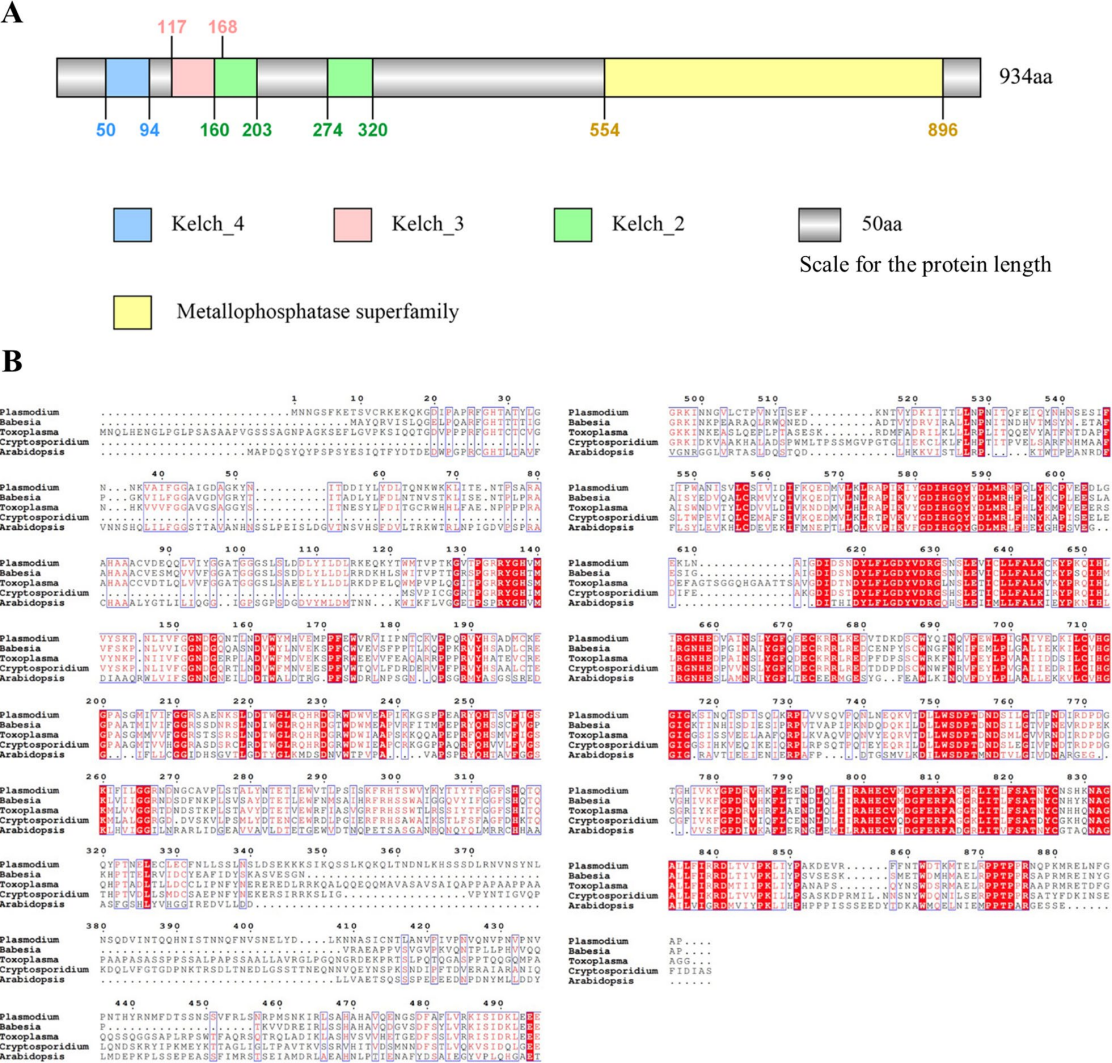
TgPPKL is a unique serine/threonine protein phosphatase protein with a kelch-repeat domain. **A** Schematic diagram of the domain architecture of TgPPKL. PPKL contains kelch-repeat domains at the N-terminus and protein phosphatase domains at the C-terminus. A scale length of 50 amino acids is shown for the protein length displayed in the schematic. **B** Alignment of PPKL amino acid sequences from different species. PPKL was retrieved from *Plasmodium* (XP_001348804.1), *Babesia* (XP_001609688.1), *Toxoplasma* (GT1 PR59771.1), *Cryptosporidium* (XP_001388090.1), and *Arabidopsis* (NP_171844.6). Red and blue rectangular areas indicate relatively conserved amino acid sites

To identify the protein phosphatase PPKL in *T. gondii*, we obtained the gene *TGGT1_290170*, herein termed TgPPKL, by comparing the protein sequence of Arabidopsis BSU1 in the TOXODB database. InterPro domain analysis showed that TgPPKL contains four conserved domains, the kelch-repeat domain at the N-terminus, and a metallophosphatase domain at the C-terminus (Fig. 1A). Alignment of the amino acid sequences of PPKL from *Toxoplasma*, *Plasmodium*, *Babesia*, *Cryptosporidium*, and *Arabidopsis* identified several regions with identical amino acid sequences, which are mainly distributed at the C-terminus of the protein (Fig. 1B). The N-terminal domain of PPKL contained tandem kelch repeats, and the protein phosphatase domain at the C-terminal was conserved (Fig. 1A, B). It contains core motifs common to the phosphorylated protein phosphatase family throughout eukaryotes, including GDxHG, GDxVDRG, RG, GNHE, and HGG (where “x” denotes any amino acid) (Fig. 1B).

**Fig. 2:**
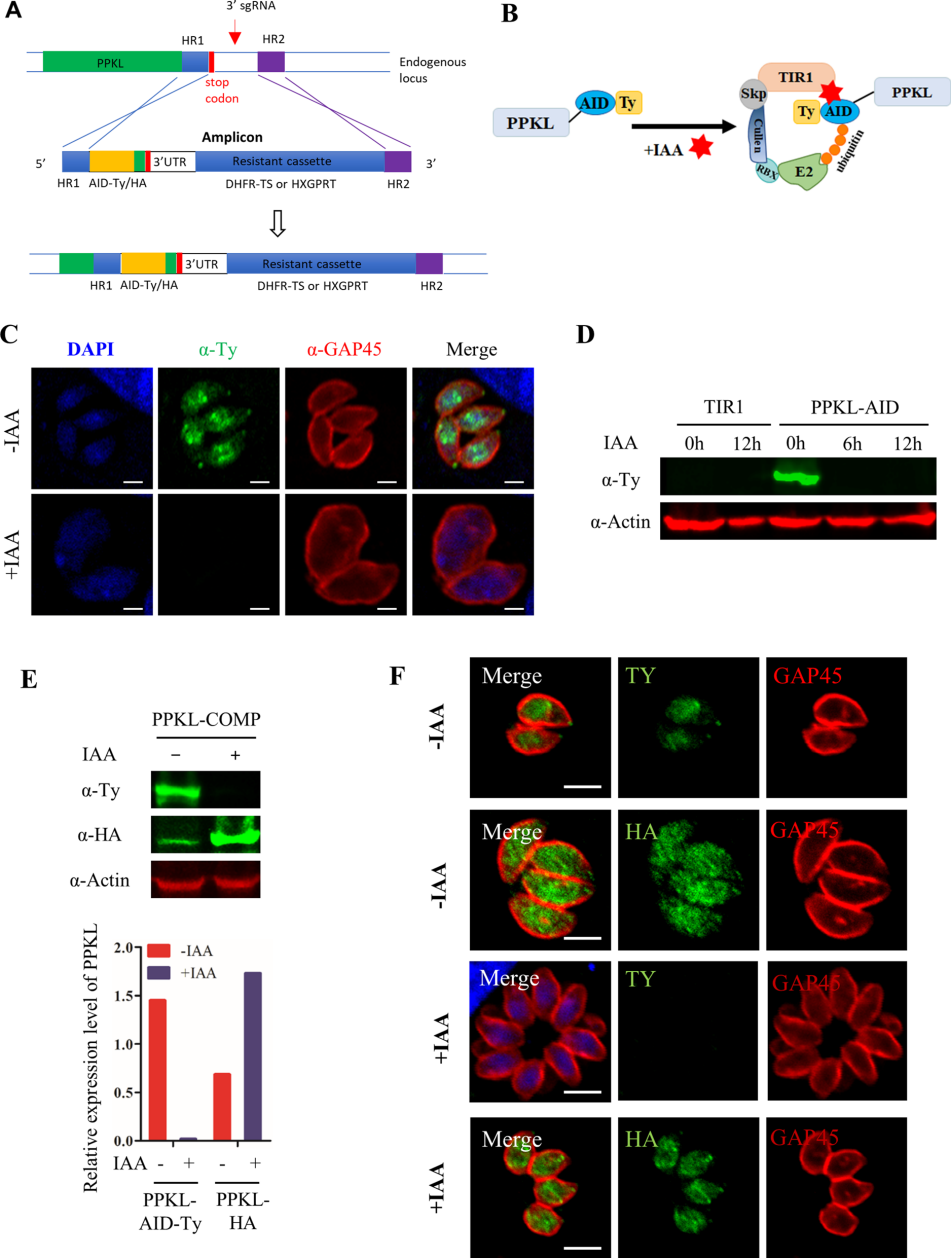
Generation and identification of TgPPKL-AID and its complementation line. **A** CRISPR-mediated C-terminal AID tagging at the endogenous locus of the 3′-terminus of *ppkl* in the RHΔ*ku80*Δ*hxgprt*/TIR1 line. The homologous regions (HR1 and HR2) derived from upstream of the stop codon and downstream of single-guide RNA (sgRNA) contain about 40 base pairs (bp) of DNA sequences, while the amplicon was amplified from a generic plasmid that contained a DNA fragment encoding an AID-Ty-3′UTR-resistant expression cassette. The amplicon and pCAS9-3′sgRNA were combined and transfected into the parasites. **B** Schematic diagram of the plant auxin-inducible degron system for the PPKL-AID fusion protein. **C**, **D** IFA and western blot detection of the PPKL-AID-6Ty fusion. The parasites were harvested for detection of the fusion protein by IFA (**C**) and western blot (**D**). GAP45 was used as the control for the IFA, while actin antibodies served as the loading control on the western blots. Scale bars = 2 μm. **E**, **F** A PPKL-COMP line was generated by transfection of a plasmid expressing PPKL-3HA. The parasites were grown for 24 h, followed by incubation in the presence or absence of auxin for 12 h. The expression was detected by western blots and IFA, where control antibodies were used for the detection. The intensity of the bands was quantified, followed by comparing the intensity of PPKL-AID-Ty and PPKL-HA with the intensity of actin, resulting in the relative expression of the endogenous and ectopic PPKL in the parasites (**E**). The parasites as described in (**E**) were analyzed by IFA using antibodies as listed in the figures. Scale bars = 2 μm

To dissect the function of PPKL, the plant auxin-induced degron (AID) system was used to conditionally downregulate the protein level of PPKL in *T. gondii*. The AID-6Ty fragment was fused at the C-terminus's endogenous locus of the *ppkl* gene using a CRISPR-Cas9 approach, as described in our previous study (Fig. 2A and Additional file 1: Tables S1–S3) [15]. A diagnostic polymerase chain reaction (PCR) analysis further confirmed the parasite line, as shown in Additional file 1: Figure S1. This genetically engineered parasite line was generated in the background of the TIR1-expressing line, in which the addition of auxin can trigger the formation of a protein complex that leads to protein degradation of AID fusions, as illustrated in Fig. 2B. By application of the TIR1-AID system, the protein level of PPKL could be conditionally regulated in the parasite. Immunofluorescence analysis (IFA) showed that PPKL-AID-6Ty was partially localized in the apical region and the cytoplasm, and appeared to be concentrated in the nucleus of the *T. gondii* parasite (Fig. 2C). The addition of auxin (IAA) led to an undetectable level of the protein in parasites grown in auxin for 24 h (Fig. 2C). To confirm the protein level of PPKL, we further analyzed the protein level by western blot after the growth of the parasites in auxin for 6 and 12 h. The analysis showed that PPKL-AID-6Ty was no longer detectable on western blots in parasites with the addition of IAA (Fig. 2D). The first observation of auxin induction in the AID line showed that the parasite body appeared enlarged after auxin induction (Fig. 2C). We then wondered whether the genetic operation was properly manipulated in the AID line. We examined the parasite line using a complementation strategy, where TgPPKL-HA was expressed from a plasmid transfected into the PPKL-AID-6Ty line, as shown on western blot (Fig. 2E). The western blot showed that the protein level appeared to be increased upon depletion of the endogenous copy of PPKL (Fig. 2E). The IFA of the complementation line demonstrated that PPKL-HA was largely distributed to the cytosol in the parasites without auxin, yet it appeared to be much clearer at the apical region in parasites grown in auxin (Fig. 2F). In addition, the parasite morphology appeared to be restored in the auxin-induced parasites (Fig. 2F), relative to the PPKL-AID grown in auxin. We noted that the ectopic expression copy of PPKL-HA in the parasites showed a slight difference in localization relative to the AID fusion. However, the apical foci and the central concentration of PPKL-HA in the parasites were evident (Fig. 2F).

### TgPPKL is essential for parasite growth in vitro and in vivo

**Fig. 3:**
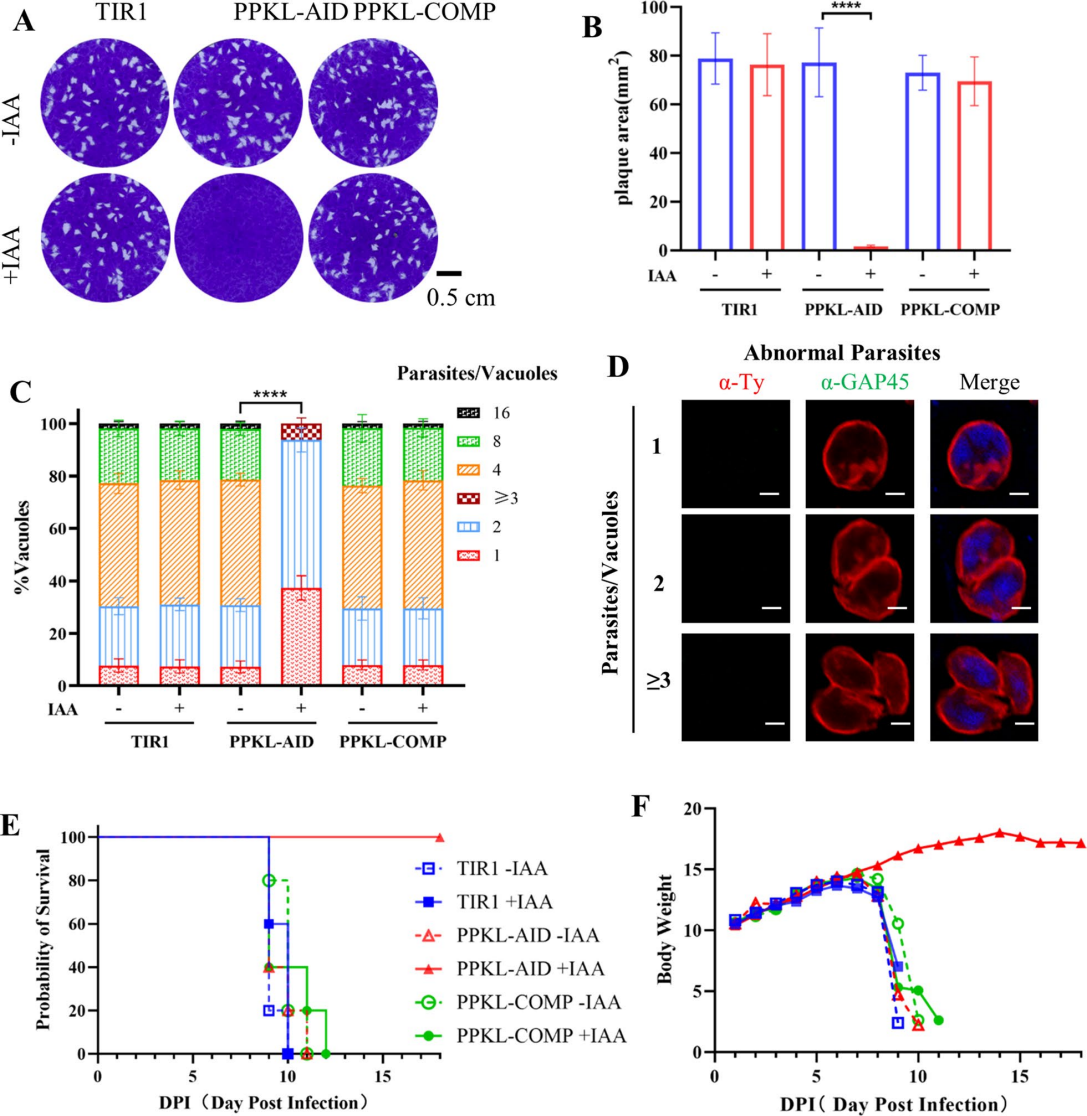
Depletion of PPKL caused strong defects in parasite growth in vitro and in vivo. **A**, **B** The lytic cycle was examined by plaque formation assay. Tachyzoites (150 parasites/well) were tested on HFF host cell monolayers and allowed to grow in the absence or presence of auxin for 7 days (**A**). Plaque areas were scored for the TIR1, PPKL-AID, and the complementation line (**B**). Three independent experiments were performed in triplicate. Scale bar = 0.5 cm. **C** Parasite replication was assayed for three of the parasite lines. Parasites were allowed to grow in the absence or presence of auxin for 24 h, followed by IFA for scoring of vacuoles containing different numbers of individual parasites. Data were analyzed by two-way ANOVA with Tukey’s multiple comparisons. ****P < 0.0001 for different numbers of parasites/vacuoles. **D** Examples of the PPKL-AID parasites grown in auxin. Parasites exhibited deformed morphology with enlarged bodies and multiple nuclei. Scale bars = 2 μm. **E**, **F** Survival of mice infected with TIR1, PPKL-AID, and the complementation line (PPKL-COMP) in the absence and presence of auxin. Each mouse was infected with 100 tachyzoites, and five mice per cage were randomly grouped and supplied with either the vehicle or auxin. Six-week-old female BALB/c mice were used in the assay, and body weight and health were monitored daily. Three experiments were performed, and an example of the experiments is shown

To investigate the effect of PPKL on the growth of *T. gondii*, we tested the ability of parasites to form plaques on human foreskin fibroblast (HFF-1) host cell monolayers. The parasite lines, including the parental line, the AID fusion (PPKL-AID), and the complementation line (PPKL-AID/PPKL-HA), appeared to form normal plaques on HFF host cell monolayers in the absence of auxin (Fig. 3A), supporting the proper operation of the plaque formation assay and the adequate generation of the PPKL-AID line. Compared with the average growth of the TIR1 line in auxin, the AID fusion parasites had no discernible plaques formed in the HFF host cell monolayers when cultured in a medium containing auxin (Fig. 3A). As expected, the complementation line was able to form regular plaques in the presence of the inducer auxin. To gain a clear view of the parasite growth results, we statistically analyzed the plaque numbers from three lines, which obviously supported the loss of plaque formation capability upon depletion of TgPPKL-AID and the complementation capability of PPKL-HA in the AID parasites (Fig. 3B). The formation of plaques by the parasite is attributed to the lytic cycle of invasion, replication, and egress of the parasites on host cell monolayers. We then wondered whether the parasite replication contributed to the defect in the lytic cycle. The parasite lines were cultured in host cells for 24 h in the absence or presence of auxin, and the parasite numbers inside the vacuoles were scored using an IFA approach. The replication assay clearly showed that the depletion of TgPPKL resulted in the accumulation of low numbers of individual parasites inside the vacuole. At the same time, the parental line and the complementation line grown in auxin appeared to have normal distributions of parasite numbers (Fig. 3C). This assay demonstrated that PPKL strongly affects the formation of individual parasites. To provide a better view of the parasite replication, here we show some images of parasites upon depletion of TgPPKL, from which we observed enlarged bodies with 2–3 parasite nuclei (Fig. 3D). This observation suggested that depletion of PPKL caused the accumulation of parasite nuclei, indicating that PPKL is involved in parasite division.

In order to investigate the effect of PPKL on parasite growth in vivo, intraperitoneal infection of mice was carried out with TIR1, the PPKL-AID line, and the complementation line. They were subsequently administered orally and intraperitoneally either vehicle or auxin on a daily basis, as reported previously [16, 17]. Both the mice receiving the vehicle and those infected with TIR1 receiving auxin died from toxoplasmosis by days 10–12 post-infection (Fig. 3E). Conversely, the mice infected with the PPKL-AID line and administered auxin ultimately escaped the lethal effect. However, the virulence loss in PPKL-AID was rescued by PPKL-HA, where the complementation restored the virulence of the parasites (Fig. 3E). Detailed data demonstrated that the body weight of the mice dropped sharply on day 7 in the groups, ultimately leading to the death of the mice. In contrast, mice infected with the PPKL-AID line receiving auxin continued to gain body weight (Fig. 3F). These results indicated that the outcomes of this study were robust and that PPKL was essential for parasite growth and virulence in mice.

### Depletion of PPKL resulted in an enlarged parasite body, parasite budding defect, and elongated cytoskeleton fibers

**Fig. 4:**
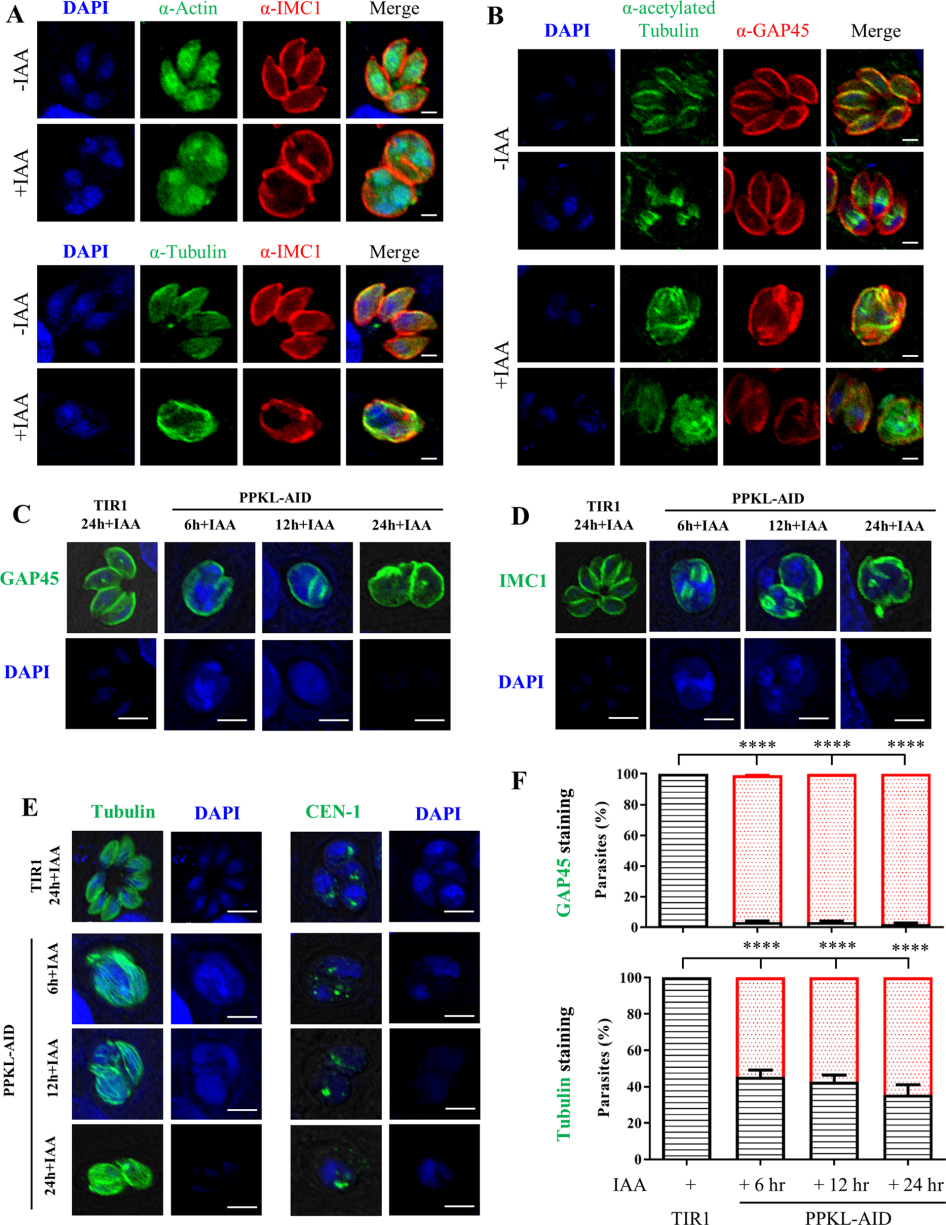
Depletion of PPKL caused defects in daughter parasite maturation and parasite morphology. **A**, **B** Parasite morphology was strongly affected by depletion of PPKL. Parasites were grown in the presence or absence of auxin for 24 h, followed by fixation for IFA with antibodies against actin, tubulin, and acetylated tubulin. IMC1 and GAP45 were used as controls for the IFA. Three independent experiments were performed, with similar outcomes. Scale bars = 2 μm. **C**–**E** The maturation of the daughter parasites and cytoskeleton integrity were strongly disrupted upon depletion of PPKL. Parasites were grown for 24 h in total, and the PPKL-AID line were induced in auxin for 6, 12, and 24 h (h). The parasites were examined by IFA using antibodies against GAP45, IMC1, tubulin, and centrin1. The parasite body was enlarged with clear emerging daughter parasites, multiple nuclei, and elongated cytoskeleton fibers in PPKL-depleted parasites. Scale = 5 μm. **F** Quantification of parasites stained with GAP45 and tubulin. The parasites observed in (**C**–**E**) were scored for the calculation of parasites with normal morphology (black column), parasites with enlarged bodies (stained by GAP45, red column), and parasites with elongated cytoskeleton fibers (stained by tubulin, red column)

As the parasites appeared to be enlarged upon depletion of PPKL, we then analyzed some of the key organelles and the cytoskeleton in the parasites using an IFA approach after induction of the parasites in auxin for 24 h. The organelles analyzed by IFA included the apicoplast (ACP), rhoptries (ROP5), endoplasmic reticulum (ER) [lipid phosphate phosphatases (LPPs)], centriole (Cen1), micronemes (MIC2), inner membrane complex (IMC1, GAP45, and MLC1), cytoskeleton (tubulin and acetylated tubulin), actin fibers (actin), and the dense granule (GRA7). In the parasites treated with auxin and processed for IFA, we observed that the parasite morphology appeared distorted, as shown by the stain with markers for the inner membrane complex and tubulin for the parasite cytoskeleton fibers (Additional file 1: Figure S2 and Fig. 4A, B). In the stain with Cen1, the parasites without IAA induction appeared to have one or two Cen1 dots per nucleus (Additional file 1: Figure S2A). In contrast, the Cen1 dot appeared to have one in some nuclei, but zero in others (Additional file 1: Figure S2A). This is likely the key phenotype in which the PPKL-depleted parasites appeared as enlarged bodies with multiple nuclei inside the bodies. However, we observed that several of the markers appeared to be localized to the expected location in the parasites, which included the apical orientation of MIC2 and ROP5, the network location of the LPPS, the anterior location of ACP, the evenly distributed actin fibers, and the cytosolic and PV distribution of GRA7. However, we observed that MIC2 tended to attach to the parasite surface in the PPKL-depleted parasites, and ROP5 appeared to be diffused in the apical region (Additional file 1: Figure S2A). These observations suggested that depletion of PPKL had strong effects on the secretory organelles, in contrast to the indiscernible appearance of the inheritable organelles (i.e., the endoplasmic reticulum [ER], the apicoplast) and the structure associated with parasite division (e.g. the centriole). In addition, PPKL is associated with the inner membrane complex and the cytoskeleton fibers (tubulin), the defects of which would cause distortion of parasite morphology.

PPKL contains several kelch-repeat domains. As the kelch-repeat domain is involved in many cellular functions, such as the formation of the cytoskeleton and transcriptional regulation [18], we then examined the effect of PPKL on actin and tubulin, as well as the acetylated form of tubulin and the extracted form of cytoskeleton tubulin in the parasites (Fig. 4A, B, and Additional file 1: Figure S2B). It has been shown that the parasite forms 22 subpellicular microtubules that define the cell shape of *T. gondii* [19] and that form a highly organized distribution in the parasite and extend from the apical cap to the parasite center but not to the basal end of the parasite [20]. The acetylated form of tubulin is required for parasite division [21]. Therefore, the examination of tubulin is expected to provide more information about the parasite morphological distortion in PPKL-depleted parasites. The analyses demonstrated the regular distribution of microtubules (stained by tubulin) at the parasite surface in the PPKL-AID parasites grown without auxin. In contrast, the subpellicular distribution of microtubules was distorted in parasites grown in auxin (Fig. 4A). The staining with antibodies against acetylated tubulin clearly showed dividing daughter parasites and newly matured parasites in the PPKL-AID grown without auxin (Fig. 4B). However, upon depletion of PPKL in the parasites, we were unable to identify budding parasites with similar staining of acetylated tubulin (Fig. 4B). Instead, a random distribution of acetylated tubulin in the enlarged bodies was observed. Based on the IFA observations, we reasoned that the parasite cytoskeleton was severely affected by the depletion of PPKL in the parasite, especially in the dividing parasite. We then attempted to observe the extracted microtubules from the parasites using treatment with deoxycholate, which is able to solubilize the parasites for extraction of the cytosokeleton. In the parasites processed for staining with tubulin, we observed an umbrella-shaped cytoskeleton in the TIR1 and the un-induced parasites. Conversely, a random distribution of tubulin fibers was easily observed in the parasites grown in auxin (Additional file 1: Figure S2B). Collectively, we identified a strong defect in the cytoskeleton in the matured parasites and the budding parasites upon depletion of PPKL.

Based on the above observations, we then wondered whether the phenotypes of PPKL-depleted parasites exhibited a similar extent of defects in an earlier induction time. To examine these phenotypes, we performed the IFA for the PPKL-AID line grown for 24 h but in auxin only for 6, 12 and 24 h. The parasites processed for these analyses clearly showed similar defects, where the PPKL-AID parasites appeared to be enlarged in auxin for 6 h, relative to the normal morphology of the TIR1 grown in auxin for 24 h, as clearly demonstrated by staining with GAP45, IMC1, Cen1, and tubulin (Fig. 4C–E). In addition, we observed the formation of daughter parasites in the mother PPKL-AID parasites grown for 6 and 12 h, as shown by staining of IMC1 (Fig. 4D). We also observed elongated cytoskeleton tubulins in the AID parasites grown for 6 and 12 h (Fig. 4E). Moreover, Cen1 stain was observed but in the absence of the nucleus by the stain in the 6-h-induced parasites; instead, the coupling of Cen1 and the nucleus was evident in the TIR1 control parasite (Fig. 4E). To gain a better view of the phenotypes, we quantified the parasite defects for the staining with GAP45 and tubulin, which supported the above observations for PPKL-depleted parasites (Fig. 4F). However, in contrast to the regular distribution of 1–2 nuclei per parasite in the parental line, the AID parasites exhibited 2–3 nuclei per parasite, further suggesting that PPKL is involved in parasite division. Taken together, the results show that the PPKL-depleted parasites are unable to form mature parasites, suggesting that PPKL is associated with parasite replication and proper organization of the cytoskeleton tubulins.

### Depletion of PPKL resulted in defects in parasite motility and egress

**Fig. 5:**
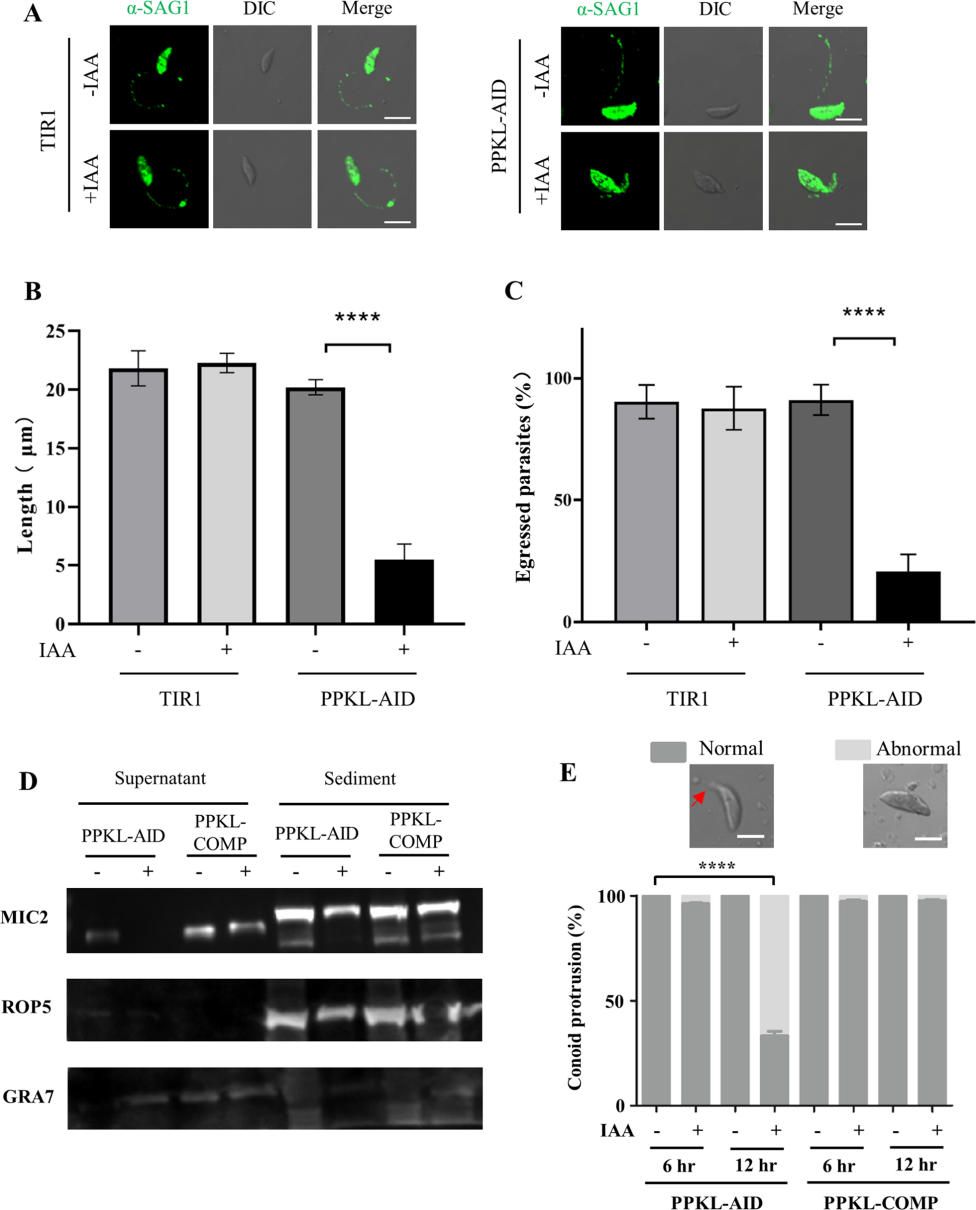
Parasite motility and egress were strongly impaired upon depletion of PPKL. Parasites were grown for 24 h in total, and induced in auxin for 6 h prior to processing for assays of parasite motility and egress. After parasite purification, extracellular parasites on poly-lysine-coated coverslips were allowed to move freely for 20 min at 37 °C, and processed for IFA using antibodies against SAG1 for visualization. For the parasite egress assay, intracellular parasites were stimulated in DMEM containing 3 μM A23187 for 5 min at 37 °C, followed by IFA using antibodies against GRA7 and GAP45. **A** Representative images of parasites with tails are shown. (**B**, **C**) Quantification of parasite motility tail lengths and egress percentages for the TIR1 and PPKL-AID lines. Data are shown with mean ± SD and analyzed by two-way ANOVA with Tukey’s multiple comparisons. Scale: 5 μm. ****P < 0.0001. **D** Depletion of PPKL resulted in blocked secretion of the microneme protein MIC2. The parasites, including PPKL-AID and the complementation line (PPKL-COMP: PPKL-AID/PPKL-HA), were grown in ±IAA for 12 h, followed by harvesting of the parasites for incubation with 1% ethanol and 1% BSA for 10 min. The parasites were subjected to centrifugation to collect the supernatant and sediment, which were examined by western blot with antibodies against MIC2, ROP5, and GRA7. **E** The parasites incubated in ±IAA for either 6 or 12 h were harvested for examination of conoid protrusion under stimulation by 3 μM A23187. Examples of the parasites with normal protrusion and abnormal appearance of the apical region are shown. The red arrowhead points to the apical region of the parasite. The parasites were scored and plotted as percentage of conoid protrusion. Data were analyzed by two-way ANOVA of Tukey’s multiple comparisons. ****P < 0.0001, scale bar: 2 μm

As PPKL is partially localized to the apical end, we wondered if the localization of PPKL has a potential link with its function in parasite motility and egress. We then analyzed parasite motility by measurement of SAG1 staining length after incubation of extracellular parasites on coverslips. The measurements demonstrated that the parasite tail length (SAG1 staining) decreased dramatically for the parasites induced for only 6 h (Fig. 5A, B). To examine parasite egress, the parasites were stimulated to egress by 3 μM A23187 for 5 min, followed by IFA staining for GRA7 and GAP45, as demonstrated in our previous study [15]. This assay showed a strong decrease in parasite egress for the PPKL-AID parasites in auxin for 6 h versus in ethanol for 24 h (Fig. 5C). These results thus confirm the strong defect in parasite motility upon depletion of PPKL. Based on these results, we further asked whether depletion of PPKL would affect the secretion of microneme proteins and protrusion of the conoid. Under stimulation of the parasites with 1% ethanol and 1% bovine serum albumin (BSA), as performed in our previous study [15, 22], the parental line was able to normally secrete the microneme marker protein MIC2 (Fig. 5D), as shown on western blots of the supernatants. In contrast, the AID line induced for 12 h had a strong defect in MIC2 secretion, which was consistent with the observations of MIC2 localization in PPKL-depleted parasites. The complementation copy of PPKL-HA indeed restored the secretion of MIC2 (Fig. 5D). In addition, our analysis showed that the conoid protrusion of extracellular parasites induced by the calcium ionophore A23187 was strongly reduced in PPKL-depleted parasites which were grown for 24 h but incubated in the presence or absence of auxin for either 6 or 12 h (Fig. 5E). Collectively, these analyses suggest that PPKL is associated with the structural stability of the apical end and microneme secretion, potentially via a link with the cytoskeleton in the apical region.

### The proximity proteomics of PPKL identifies potential interacting proteins

**Fig. 6:**
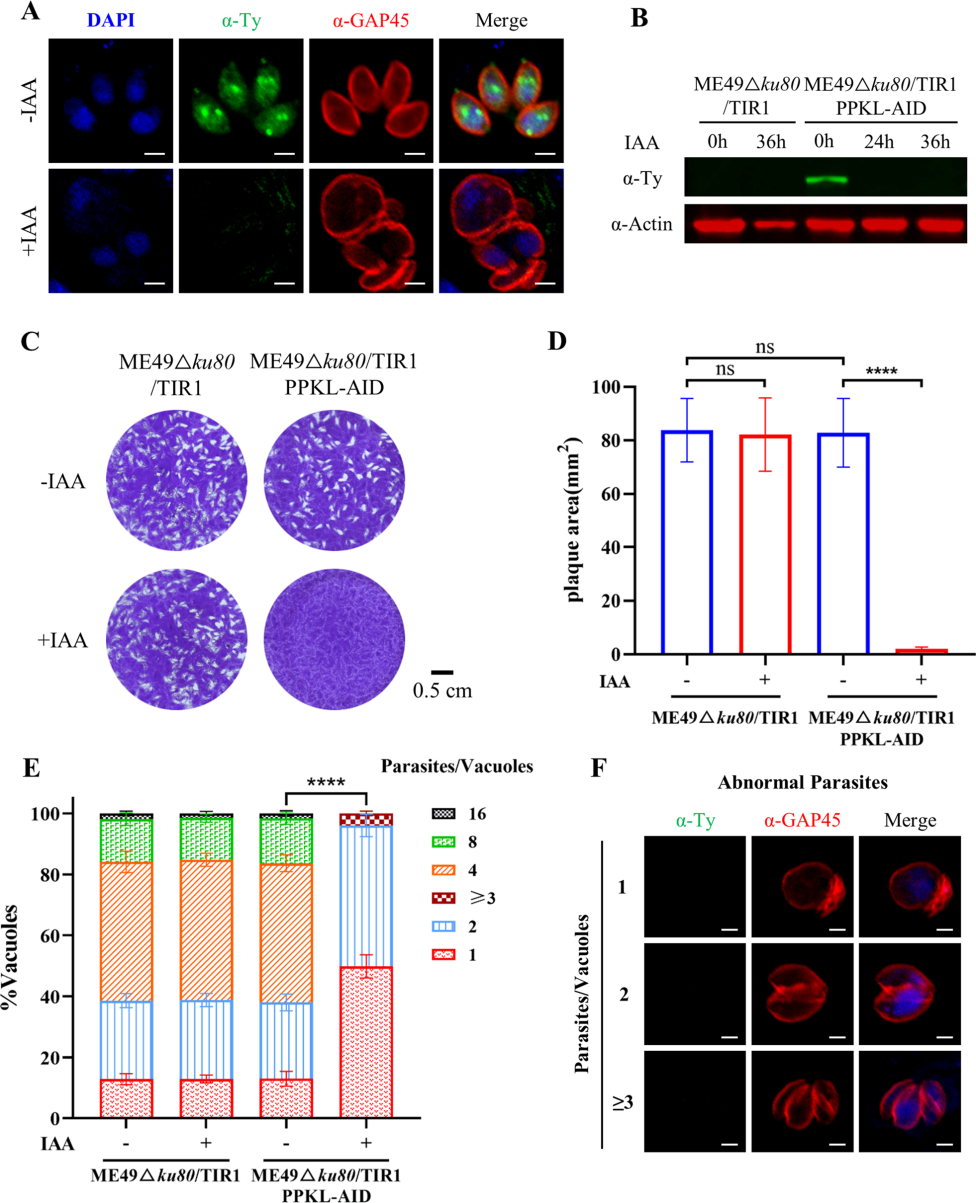
PPKL is essential for parasites in the type II strain ME49. **A**, **B** PPKL was efficiently depleted by the addition of auxin. The PPKL-AID line was grown in auxin for 24 h, followed by IFA using antibodies against the Ty epitope and GAP45. Western blotting was performed for parasites induced in auxin for the indicated times, and actin served as the loading control. Scale bars = 2 μm. **C**, **D** Parasite growth was examined by plaque formation assay. The parasites were grown on HFF host cell monolayers for 10 days, followed by fixation and staining. Three independent experiments were performed in triplicate. The plaque areas were scored using ImageJ software, and data were expressed as mean ± SD and analyzed by two-way ANOVA with Tukey’s multiple comparisons. ****P < 0.0001. Scale = 0.5 cm. **E**, **F** Depletion of PPKL resulted in defects in parasite replication and morphology. Parasites were grown in ±IAA for 24 h, followed by IFA using antibodies against GAP45 for imaging and scoring of parasites in vacuoles (**E**). At least 200 vacuoles were scored for each replicate, and three independent experiments were performed in triplicate. Vacuoles with 1–3 parasites are shown from the IFA of parasite replication (**F**). Scale = 2 μm. ****P < 0.0001

As predicted, PPKL contains kelch-repeat domains. These domains can mediate protein–protein interactions [23]. To identify the potential interacting proteins, we utilized a proximity biotin labeling approach which can label proximal proteins within 10–30 nm around the bait, as demonstrated to define the protein interaction network at the conoid and the micropore in our previous studies [24, 25]. A TurboID-PPKL strain was then constructed at the N-terminus using CRISPR/Cas9 gene editing technology in a *T. gondii* parental line. The IFA showed strong staining of the parasite central region, but with fairly strong diffusion of the PPKL protein. In addition, the apical complex staining appeared to be evident in some of the parasites (Additional file 1: Figure S3A). The TurboID biotin ligase activity was examined by adding biotin and by detecting biotinylated proteins using streptavidin reagents on western blots and IFA. The IFA stain showed a huge fluorescent region in the center of the parasites, an apical region, and clear diffusion in the parasites (Additional file 1: Figure S3B), which suggested that the TurboID fusion was functional. The biotinylated proteins in the parasites were confirmed by western blots (Additional file 1: Figure S3C), thus supporting the strong labeling activity of the TurboID-PPKL fusion (Fig. 6B, C). Samples extracted from the TurboID and parental lines were subjected to mass spectrometry. The resultant mass spectrometry datasets (*N* = 2 for each) were analyzed by comparing the peptide numbers retrieved from the PPKL-TurboID fusion with those from the parental line (Fig. 5D). This analysis showed that PPKL had the highest *P*-value associated with its enrichment in the TurboID line, as expected for a bait protein.

The candidates selected by the analysis with *P*-value < 0.05 were further subjected to protein–protein interaction analysis using STRING online software (https://string-db.org/). This analysis identified six potential interacting proteins with PPKL (Fig. 5E), which included protein kinase, myosin A, PP2C, DrpB, Hsp90, and peptidyl-prolyl cis–trans isomerase (Fig. 5F). In addition, the labeling approach identified six hypothetical proteins. These results provided us with a potential link between PPKL and diverse types of proteins. However, the results suggest that these potential interacting proteins are substrates of PPKL phosphatase. The proteomic candidates with significant differences (*P* < 0.05) were further subjected to Gene Ontology (GO) enrichment analysis, which showed that those proteomic hits were enriched in diverse metabolic processes (Fig. 5G). This GO enrichment analysis will likely provide additional information about the strong defect in parasite replication and morphology upon depletion of PPKL. Collectively, the strong defect in parasite replication is likely to be associated with the potential and diverse substrates of PPKL on protein–protein interaction and substrate dephosphorylation.

### PPKL is required for parasite morphology in a type II parasite strain ME49

We were further motivated to analyze the phenotypic effect of PPKL depletion in another type of *T. gondii* strain ME49 using the plant AID system [17]. The ME49 strain has a medium level of parasite growth and virulence in host cells relative to the RH strain. The phenotypic analysis in this strain would provide additional information about PPKL in *T. gondii*. We generated the parasite line PPKL-AID using the CRISPR-Cas9 approach. In ME49, we observed a similar localization of PPKL by staining of PPKL-AID fusion to the protein in the RH background, where PPKL is partially localized to the apical complex, the nucleus, and cytosolic puncta inside the parasite (Additional file 1: Figure S3A). In the parasites grown in auxin, we observed the clear disappearance of the AID fusion (Fig. 6A). The depletion of PPKL-AID by auxin was further confirmed by western blot, suggesting that the TIR1-AID system was highly efficient and specific to the AID fusion (Fig. 6B). We then analyzed the growth phenotype on plaque formation and parasite replication, from which no discernible plaques formed in host cell monolayers, and a defect in parasite replication was observed (Fig. 6C–E).

**Fig. 7:**
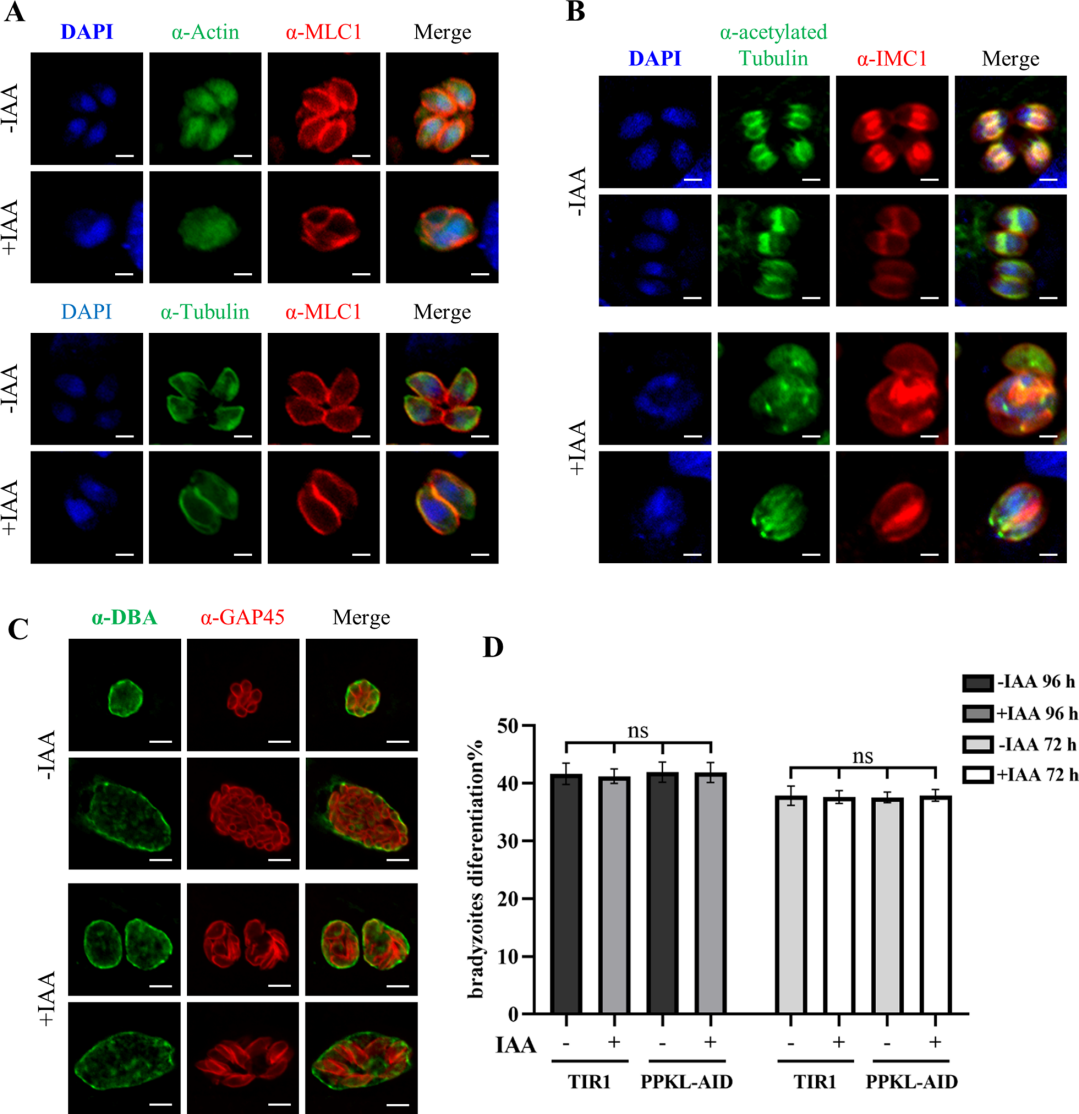
Depletion of PPKL resulted in defects in parasite replication and morphology in the type II strain ME49. **A**, **B** Parasites were grown for 24 h in the absence or presence of auxin, followed by IFA using antibodies against MLC1, IMC1, actin, tubulin, and acetylated tubulin. The antibodies against MLC1 and IMC1 served as the controls for the IFA. Scale bar = 2 μm. **C**, **D** Parasites with depleted PPKL were not associated with differentiation of ME49. Parasites were grown on HFF in 1% FBS in pH8.1 μm

Similarly, we observed enlarged parasite bodies that appeared spherical or distorted (Fig. 6F). Based on the growth phenotype, we further analyzed the cytoskeleton in the parasites by IFA. In the parasites grown in auxin, we could not observe microtubule fibers inside the parasites, in contrast to the parasites without auxin induction (Fig. 7A). The acetylated tubulin inside the parasite body appeared to diffuse but not to concentrate in the daughter parasites, although a tubulin dot was observed to be located at the apical region of the daughter parasites (shown by the IMC1 staining) in parasites grown in auxin (Fig. 7B). Once again, in the type II strain ME49, we observed defects in acetylated tubulin in daughter parasites and parasite morphology in PPKL-depleted parasites.

As the ME49 strain tends to differentiate into bradyzoites, we then examined the parasite lines by growth in a pH 8.1 medium, and tested the bradyzoite form by a fluorescent reagent Dolichos biflorus agglutinin (DBA). After treatment of the parasites in the alkaline medium for 72 and 96 h, the PPKL-AID line grown in the presence or absence of auxin was fully capable of differentiating into the bradyzoite form, as detected by the DBA stain and quantification of the DBA-stained vacuoles (Fig. 7C–D). The fluorescence of DBA was easily observed in vacuoles that contained individual parasites with clearly distorted morphology (Fig. 7C).

## Discussion

The balance between phosphorylation and dephosphorylation is finely regulated by protein kinases and phosphatases and is critical for almost all biological processes. Interestingly, some protein kinases and phosphatases are possessed by apicomplexans and green plants but not by mammals. This may be because apicomplexans possess a set of plant-like genes, which may be descended from their common photosynthetic ancestor [25], and which is consistent with the theory that non-photosynthetic eukaryotes and red algae endosymbiotically form apicomplexans. Since the corresponding genes do not exist in mammals, the proteins encoded by these plant-like genes are considered potential targets for the intervention of apicomplexan protozoa. In contrast to *Arabidopsis* BSU1 [13], which was localized only to the nucleus, *Plasmodium* PPKL was localized not only to the apical region, the nucleus, but also to the cytoplasm. Similarly, *Toxoplasma* PPKL was localized to the apical region and to the nuclei and cytoplasmic puncta. The apical complex plays a vital role in *Toxoplasma* invasion, egress, and gliding motility [25, 26], and expectedly, depletion of PPKL disrupted the stability of the apical cytoskeleton, the parasite motility, and the parasite egress. Meanwhile, the depletion of PPKL was observed to cause defects in the maturation of daughter parasites and parasite morphology.

The plant-like protein phosphatase PPKL, a member of the serine/threonine protein phosphatase family, contains two conserved domains: the N-terminal kelch-repeat domain and the C-terminal protein phosphatase domain. Among the apicomplexans, a PPKL with three to four Kelch-like domains is present in the genomes of *Plasmodium*, *T. gondii*, and *Cryptosporidium* [27]. Sequence analysis revealed high conservation of PPKL sequences in *Plasmodium*, *Toxoplasma*, *Cryptosporidium*, and *Babesia*, which may indicate a common requirement for this unique protein phosphatase. The function of PPKL in apicomplexan parasites has only been studied in *Plasmodium*. The results showed that PfPPKL affects the integrity of the apical morphology and regulates the differentiation, motility, and invasion of the ookinete [14]. Proteins containing kelch-repeat domains are known to be involved in many cellular functions, particularly cytoskeleton formation and transcriptional regulation [18]. Therefore, we examined the effects of PPKL on *Toxoplasma* actin and tubulin by IFA, which demonstrated a strong defect in the cytoskeleton in parasites with depleted PPKL. In the parasite, the microtubules extend down the parasite periphery from the apical ring to the half length of the parasite. The depletion of PPKL resulted in a strong defect in the cytoskeleton tubulins. This phenotype was reinforced by staining the microtubules in parasites extracted with deoxycholate. In addition, we observed a defect in daughter parasites with acetylated tubulins, which was shown to be involved in parasite division in a previous report [21]. Therefore, it is not surprising that the PPKL-depleted parasites exhibited a strong defect on parasite replication, as shown by the presence of multiple nuclei in one mother parasite.

The kelch domains form a β-propeller tertiary structure usually involved in protein–protein interactions [18]. We attempted to analyze potential protein–protein interactions using a proximal labeling approach with TurboID, which was used to identify interacting proteins in the conoid and in the micropore in our previous studies [24, 25]. However, it must be noted that TurboID labels not only interacting proteins but also proximal proteins that cannot interact with the bait. In our case, by combining TurboID, fold change analyses, and STRING analysis, we identified potential interacting proteins, including several proteins that have been functionally analyzed previously and some unknown proteins. However, TurboID-PPKL fusion appeared to have a strong diffusion in the parasite cytosol, except that the regular localization of the apical region and the nucleus was observed. This should explain why some unexpected components, such as ribosomal proteins and IMC-localized myosin A, were identified as top hits in the biotinylated proteins datasets. Nevertheless, proteomic analysis of TurboID-PPKL was likely to provide additional information about the potential interacting proteins for the essential nature of the protein in the parasite.

In summary, the plant-like protein phosphatase PPKL is essential for parasite replication and morphology formation in *T. gondii*. Our study confirmed that the plant-like protein phosphatase PPKL is localized in the apical complex and nucleus, and demonstrated that depletion of PPKL resulted in a significant decrease in parasite replication, along with morphological abnormalities and disordered arrangement of the cytoskeleton. In addition, PPKL is likely to interact with diverse proteins and regulate various biological processes, including metabolic processes, in parasites. Therefore, this study provided excellent insights into the function of PPKL in the most frequent human parasite and demonstrated the attractive features of PPKL for future drug development.

## Methods

### Parasite and host cell culture

Parasite lines RHΔ*ku80*Δ*hxgprt*, RHΔ*ku80*Δ*hxgprt*/TIR1, and ME49Δ*ku80*/TIR1 and their derivative lines (Additional file 1: Table S1) were grown on HFF-1 cells (purchased from ATCC [American Type Culture Collection]) in Dulbecco's modified Eagle medium (DMEM, purchased from Thermo Fisher Scientific) supplemented with 5% heat-inactivated fetal bovine serum (FBS, purchased from Gibco), two mM glutamine, and 100 units penicillin–streptomycin (purchased from Solarbio Biotech) (D5 medium) at 37 °C with 5% CO_2_. These parasite lines were used as parental lines to construct gene-edited parasite lines. TIR1 and its derived AID lines were cultured in HFF with 500 μM auxin (+IAA) or 0.1% ethanol (−IAA) for phenotypic assays.

### Antibodies and chemicals

Primary antibodies including rabbit anti-HA (Thermo Fisher Scientific, no. 71-5500), mouse anti-HA (BioLegend, no. 901501), and anti-acetylated tubulin (Thermo Fisher Scientific, no. T7451) were commercially available, while the primary antibodies including rabbit anti-ACP, rabbit anti-actin, mouse anti-IMC1, rabbit/mouse anti-GAP45, rabbit anti-tubulin, rabbit-Cen1, and rabbit anti-IPTase were generated in our own laboratory in our previous study [24, 28]. Antibodies anti-SAG1, anti-ROP5, anti-MIC2, and anti-GRA7 were generous gifts from Prof. David Sibley, and mouse anti-Ty (BB2) was a generous gift from Prof. Philippe Bastin. Secondary antibodies, including antibodies conjugated with Alexa Fluor (488 or 568) and anti-rabbit antibodies conjugated with Alexa Fluor (488 or 568), were purchased from Thermo Fisher Scientific. Fluorescent reagents conjugated with LI-COR 680CW and 800CW, and fluorescent reagents conjugated with streptavidin (Streptavidin Li-COR 800CW and Streptavidin Alexa Fluor 488) were purchased from LI-COR Biosciences, while streptavidin magnetic beads were purchased from Pierce. Chemicals included 3-indoleacetic acid (IAA/auxin) (Sigma-Aldrich, no. I2886), mycophenolic acid (Sigma-Aldrich, no. M3536), 6-xanthine (Sigma-Aldrich, no. X4002), calcium ionophore A23187 (Sigma-Aldrich, no. C7522), D-biotin (Sigma-Aldrich, no. B4639), and pyrimethamine (Sigma-Aldrich, no. 46706).

### Bioinformatics analysis of PPKL

Using the *Arabidopsis thaliana* BSU1 sequence (RefSeq: NP_171844.6) as a seed, the database for the gene and protein information of the protein phosphatase PPKL of *T. gondii* was searched in ToxoDB. The domains of *Toxoplasma* protein phosphatase PPKL were predicted using the InterPro website. ClustalW (https://www.genome.jp/tools-bin/clustalw) and ESPript (https://espript.ibcp.fr/ESPript/cgi-bin/ESPript.cgi) were used for amino acid sequence alignment of protein phosphatase PPKL retrieved from different species of *Toxoplasma*, *Plasmodium*, *Cryptosporidium*, *Babesia*, and *Arabidopsis*.

### Construction of CRISPR/Cas9 plasmids

The CRISPR/Cas9 plasmids were used for the generation of gene conditional knockdown and gene tagging. The FASTA genomic sequence of TgPPKL (−1000 bp to +1000 bp from the translation stop codon) was obtained by searching in ToxoDB (https://toxodb.org/toxo/app/). Approximately 250 bp of the sequence before the start codon or after the stop codon was analyzed in EuPaGDT (http://grna.ctegd.uga.edu/) to search for the best targets of CRISPR-sgRNA. The sgRNA sequence targeting the gene of interest (GOI) was inserted into the CRISPR/Cas9 plasmid using PCR amplification and plasmid construction with a Basic Seamless Cloning & Assembly Kit. The primers used for generation of those plasmids are listed in Additional file 1: Table S2. The plasmids were verified by sequencing using the universal primer M13R.

### Generation of the AID fusion line

The AID fusion was generated in the parental line using the CRISPR-Cas9 approach, as described previously [15, 29]. Briefly, the pLinker-AID-6Ty-DHFR plasmid, generated in our previous study [24], was used as a template to obtain an amplicon for integration of the AID-Ty cassette at the targeted gene in the *T. gondii* genome. The combination of the amplicon and the gene-specific CRISPR-Cas9 plasmid was transfected into RHΔ*ku80*Δ*hxgprt*/TIR1 or ME49Δ*ku80*/TIR1, allowing integration of the cassette at the C-terminus of PPKL. The strain was selected by the corresponding drugs (pyrimethamine), confirmed by diagnostic PCR using the F5 and R5 (Additional file 1: Table S3) primers, and further tested by IFA and western blot.

### Generation of the complementation line in the AID fusion parasite

The pHA-TgPPKL-HXGPRT complementation plasmid was constructed by the fusion of two fragments amplified from different plasmids. The plasmid pNL-HA-AID-HXGPRT, which was generated in our previous study [24], was used as a template to amplify one fragment, which contains the plasmid backbone, while another fragment was amplified from the *T. gondii* complementary DNA (cDNA) to obtain the PPKL coding sequences. The primers are listed in Additional file 1: Table S3. The complementation plasmid was then constructed using a Basic Seamless Cloning & Assembly Kit to combine the different fragments. After the complementation plasmid was obtained, the plasmid was verified by PCR using primers F9 and R9, and the subsequent DNA sequencing. The pHA-TgPPKL-HXGPRT plasmid was directly transfected into the PPKL-AID-Ty line and selected using the corresponding drugs (mycophenolic acid and xanthine).

### Indirect immunofluorescence assay

Indirect IFA was used to identify gene-edited parasite lines, localization of proteins, and replication of *T. gondii*. Parasites grown in HFF monolayers on coverslips were fixed in phosphate-buffered saline (PBS) containing 4% paraformaldehyde, followed by permeabilization with PBS containing 2.5% BSA and 0.25% Triton X-100. The parasites were incubated with different combinations of primary antibodies for 40 min, followed by incubation with the corresponding secondary antibodies conjugated with Alexa Fluor 488 or 568 for 30 min. The parasites were then washed five times, each for 5 min, with PBS containing 0.05% Tween 20 and 2.5% BSA, followed by DAPI staining and mounting medium. The parasites were then visualized under a Nikon Ni-E C2+ microscope equipped with a DS-Ri2 microscope camera, and analyzed using the Ni-E software system installed in NIS-Elements AR (Advanced Research).

### Deoxycholate extraction

Parasite lines were cultured in HFF with 500 μM auxin (+IAA) or 0.1% ethanol (−IAA). Freshly egressed or mechanically egressed parasites were attached to poly-L-lysine-coated coverslips and treated with 10 mM deoxycholate (DOC) for 20 min at room temperature. Parasites were fixed in cold methanol for 8 min, and then IFA was performed as described above. Anti-rabbit tubulin was used as the primary antibody, and Alexa Fluor 488 goat anti-rabbit was used as the secondary antibody.

### Western blot

Parasite lines were cultured in HFF with 500 μM auxin (+IAA) or 0.1% ethanol (−IAA) for 36–48 h. Freshly or mechanically egressed parasites were harvested by filtration through 3.0-micron polycarbonate membranes and resuspended in PBS with 5× Laemmli SDS sample buffer. The protein samples were boiled in a metal bath at 100 °C for 10 min, separated by sodium dodecyl sulfate–polyacrylamide gel electrophoresis (SDS-PAGE), and blotting using a Bio-Rad wet-blotting system. The membranes were incubated with different combinations of primary antibodies, followed by incubation with the corresponding secondary antibodies conjugated with LI-COR 800CW or 680CW reagents (purchased from LI-COR). Streptavidin LI-COR 800CW (purchased from LI-COR) was used to detect biotinylated proteins. The membranes were visualized using a Bio-Rad ChemiDOC MP imaging system.

### Plaque formation

Parasite lines were grown on HFF in six-well plates with 500 μM auxin (+IAA) or 0.1% ethanol (−IAA) in a D5 medium at 37 °C for 7 days (RH derivatives) or 10 days (ME49 derivatives). The host cell monolayers and parasites were fixed in 70% ethanol for 10 min, followed by staining with 0.5% crystal violet for 5 min. The plates were washed, dried at room temperature, and scanned with an HP Scanjet G4050.

### Parasite replication assay

Parasite lines were grown in HFF monolayers on coverslips with 500 μM auxin (+IAA) or 0.1% ethanol (−IAA) in a D5 medium at 37 °C for 24 h. The host cell monolayers and parasites were fixed with 4% paraformaldehyde, followed by permeabilization with 0.25% Triton X-100 in PBS and IFA using GAP45 polyclonal antibodies and secondary antibodies conjugated with Alexa Fluor 488 or 568. The parasites were observed and visualized under a Nikon Ni-E C2+ microscope equipped with a DS-Ri2 microscope camera. At least 200 parasitophorous vacuoles were analyzed for each replicate, and three independent experiments were performed in triplicate.

### Parasite motility assay

Parasites were grown for 24 h but in the presence or absence of auxin for 6 h, prior to harvesting for extracellular parasites. The purified parasites in suspensions were placed on poly-lysine-coated coverslips, followed by incubation at 37 °C for 20 min to allow the parasites to glide. The parasites were then fixed in a 4% paraformaldehyde solution and processed for IFA staining using antibodies against SAG1. The SAG1 tails and parasites were visualized and captured under a Nikon Ni-E C2+ microscope equipped with a DS-Ri2 microscope camera. The lengths of the SAG1 tails were measured, and at least 200 parasites were randomly selected for the measurements in each replicate. Three independent experiments were performed in triplicate.

### Secretion of micronemal MIC2 and parasite conoid protrusion

Micronemal secretion assay was performed according the general protocol used in our previous study [15]. In brief, the parental, AID fusion, and complementation lines were grown for 24 h but incubated in the presence or absence of auxin for 12 h. The parasites were harvested and resuspended for secretion using 1% BSA and 1% ethanol at 37 °C for 10 min. The supernatant and pellets were collected by centrifugation, followed by analysis of samples on western blots using antibodies against MIC2. The antibodies against GRA7 and ROP5 were used as controls in the assay.

The parasite conoid protrusion assay was performed using a protocol of stimulation by 3 μM A23187, as described in a previous study [15]. Briefly, the parasites grown in ±auxin for either 6 or 12 h were harvested for incubation in 3 μM A23187 on coverslips. The samples were incubated at 37 °C for 10 min, followed by fixation and permeabilization. The coverslips were then mounted in ProLong Gold Antifade Mountant with DAPI, followed by visualization under a Nikon Ni-E C2+ microscope equipped with a DS-Ri2 microscope camera. At least 100 parasites were scored for each replicate, and 2–3 biological experiments were performed in triplicate.

### Parasite egress assay

Parasites were grown for 24 h but in the presence or absence of auxin for 6 h prior to the parasite egress assay. Fresh DMEM medium was added to the parasite culture plates and incubated in a water bath at 37 °C, followed by the addition of 3 μM A23187 (final concentration) and incubation of the mixture at 37 °C for 5 min. After fixation with 4% cold paraformaldehyde, the parasites were processed with IFA using antibodies against GRA7 and GAP45, as described in our previous study [15]. GRA7 staining is able to show the vacuoles due to its localization at the PV, while GAP45 stains the parasite surface. The parasites were visualized and captured by a Nikon Ni-E C2+ microscope equipped with a DS-Ri2 microscope camera. At least 200 vacuoles were scored for each replicate, and three independent experiments were performed in triplicate.

### In vitro differentiation

Differentiation into bradyzoites was tested using culture in Roswell Park Memorial Institute (RPMI) 1640 medium (pH 8.1) in the absence of CO_2_ at 37 °C, as described previously [30]. After infection of the parasites in D5 for 3 h, the medium was replaced with RPMI 1640 medium (pH 8.1). The parasites were allowed to grow in the absence of CO_2_ at 37 °C for 72 and 96 h, followed by fixation and processing for IFA using antibodies against GAP45. The parasites were then incubated with anti-rabbit secondary antibodies conjugated with Alexa Fluor 568 and fluorescein isothiocyanate (FITC)-conjugated DBL. The parasites were then mounted for visualization using a Nikon Ni-E C2+ microscope equipped with a DS-Ri2 microscope camera. Parasite vacuoles fully or partially stained with DBL were scored as bradyzoite-positive, in comparison with the total number of vacuoles detected by GAP45 staining. Three independent experiments were performed in triplicate, and > 200 vacuoles were counted in each replicate.

### Parasite growth assay in mice

Parasite lines were used to inoculate female BALB/c mice (6-week-old) intraperitoneally, with 100 parasites per mouse [24]. After parasite infection, the mice were randomly divided into the IAA-supplied group (+IAA group) and the control group (−IAA group), with five mice per cage. The mice in the +IAA group were intraperitoneally injected with 15 mg/ml IAA daily and supplied drinking water containing 1 mg/ml IAA. The mice in the −IAA group received the same drinking water without IAA and were intraperitoneally injected with PBS in parallel. The body weight and health status of the mice were monitored daily. The mouse experiments were conducted according to the guidelines and regulations issued by the Veterinary Office of the China Agricultural University (Issue No. AW11402202-2-1).

### Generation of TurboID-PPKL strain and purification of biotinylated proteins

The TurboID-PPKL strain was generated using the CRISPR/Cas9 gene editing technique. The pNL-TurboID-Ty-DHFR plasmid, which was generated in our previous study [24], was used as a template, and the primers PPKL-M and PPKL-NL (Additional file 1: Table S3) were used to amplify the amplicon that contained the homologous fragments (DHFR-Ty-TurboID) and the TurboID fragment, and the resistant cassette. The combination of the amplicon and the gene-specific CRISPR-Cas9 plasmid was transfected into RHΔ*ku80*Δ*hxgprt* for targeting the endogenous locus of PPKL. The strain was selected using the corresponding drugs (pyrimethamine), followed by diagnostic PCR using primers F6 and R6 (Additional file 1: Table S3). The engineered parasite lines were further confirmed by IFA and western blot. The purification of biotinylated proteins was performed as described previously [31]. Briefly, parasites were completely lysed in a buffer containing 1% Triton X-100, 0.2% SDS, and 0.5% deoxycholate and sonicated using a microtip in 550 sonic dismembrators (Thermo Fisher Scientific). Biotinylated proteins were then purified using streptavidin beads (purchased from Pierce).

### Mass spectrometry and proximity proteome data analysis

Purified biotinylated proteins were eluted with SDS sample buffer containing 2 µM biotin at 90 °C for 10 min and run on SDS-PAGE gels. The protein gels were fixed and stained with Coomassie blue R250, 45% methanol, and 10% glacial acetic acid and faded with 25% methanol and 8% glacial acetic acid. The protein lanes were cut off, frozen, and vacuum-dried. The dried gels were rehydrated and finely chopped, and then the stain and SDS were removed by reduction, alkylation, and washing. The samples were digested by trypsin, dried, and redissolved in 2.5% acetonitrile and 0.1% formic acid. Each sample digest was injected into a Q Exactive HF mass spectrometer running by nanoscale liquid chromatography–tandem mass spectrometry (nanoLC–MS/MS) using a 2-h gradient on a 0.075 × 250 mm C18 column [31]. The results of protein mass spectrometry were searched in UniProt, and the *Toxoplasma* proteins were selected. Preliminary screening was then performed under the condition that the peptide of the TurboID-PPKL group was upregulated ≥ 2 (relative to the RH control group), and the differential proteins were identified according to the *t*-test. The STRING database (https://www.string-db.org) was used to analyze the protein–protein interaction network between the differential proteins, and the minimum interaction score was set as 0.4. Gene Ontology enrichment was subsequently performed, and the *P*-value cutoff was set to 0.05.

### Statistical analysis

Statistics were analyzed in GraphPad Prism 8. One-way or two-way ANOVA with Tukey’s multiple comparisons was used for normally distributed data, and one-way ANOVA with Dunnett’s multiple comparisons was used for small amounts or non-normally distributed data (*P* < 0.05 was considered significant). Experiment-specific statistical information is provided in the figure legends or associated method details, including replicates (*n*), trials (*N*), and statistical tests performed.

### Supplementary Information

**Additional file 1: Figure S1.** Diagnostic PCR of the PPKL-AID line. The integration of the AID fragment at the endogenous locus was assayed by PCR, which showed the absence of the fragment in the parental line (1) but the presence of the fragment in the AID fusion line (2). **Figure S2.** Organelle stability assay in the PPKL-AID parasites grown in auxin. (A) Parasites were grown in the presence or absence of auxin for 24 h, followed by IFA using organelle protein markers ACP for the apicoplast, ROP5 for the rhoptries, IPT for the endoplasmic reticulum, Cen1 for the centriole, MIC2 for the micronemes, and GRA7 for the dense granule. The protein markers GAP45 and IMC1 were used to label the parasite outline for observing the morphology. Three independent experiments were performed, with similar outcomes. (B) Extracted cytoskeleton in the parasites. Parasites were grown in the absence or presence of auxin, followed by extraction of the cytoskeleton by deoxycholate treatment and tubulin detection. Scale bars = 2 μm. **Figure S3.** Potential interacting proteins identified by TurboID. (A–C) IFA and western blot detection of the TurboID-PPKL fusion line. (A) The parasites were grown for 24 h and processed for IFA using antibodies against Ty and GAP45 for detection of the fusion protein. (B–C) Detection of biotinylated proteins in the TurboID-PPKL line grown in the absence (−) or presence (+) of biotin (500 μM) for 1 h. Fluorescent streptavidin reagents were used to detect biotinylated proteins in the parasite line. Actin served as the control for western blot. (D) Analysis of differential candidates identified in the proximity proteome of PPKL. The volcano plot depicts fold changes in the proteome by comparing the hits in the TurboID with those in the parental line. The *P*-values and fold changes were analyzed and plotted by log10 and log2, respectively. The heat map is shown for the log10 of *P*-values. The hits with *P*<0.05 were labeled with numbers listed by sequential orders in the table (Table S4). (E–F) The interaction of differential protein candidates retrieved from (D) was predicted using the STRING database, and the sequential order numbers were listed in the interacting network (E). The candidates in the core interactome of PPKL were listed with information on CRISPR fitness scores(34), the sequential order, and known information published. (G) Gene Ontology analysis of the 28 proteins with *P*<0.05. The hits were identified by statistical analysis in (D) and uploaded for GO analysis in ToxoDB. **Table S1.** Lines used in this study. **Table S2.** Plasmids used in this study. **Table S3.** Primers used in this study.

**Additional file 2: Table S4.** Statistical analysis of mass spectrometry datasets identified in the purified samples from the parental line and the TurboID-PPKL line using streptavidin beads. The fold changes and *P*-values were calculated by comparing the peptide numbers of the TurboID-PPKL (PPKL_1/2) with those of the parental line (RH_1/2). Note that the peptide number with zero in the RH was considered as 1 for the analysis. The results were used to plot Figure S3D, and the hits with *P*<0.05 were used for protein interaction analysis by STRING (Figure S3E) and Gene Ontology analysis in ToxoDB (Fig S3G).

## Data Availability

The minimally processed datasets of proteomics are shown in Additional file 2: Table S4. The scaffold file directly exported from the raw data has been deposited in the OMIX, China National Center for Biotechnology/Beijing Institute of Genomics, Chinese Academy of Sciences (accession number: OMIX005129). *Toxoplasma gondii* genome information can be found in ToxoDB release 53 (http://toxodb.org), and eukaryotic pathogen, vector, and host information resources can be found in VEupathDB (http://veupathdb.org).
