# Supplementary material for: The plant-like protein phosphatase PPKL regulates parasite replication and morphology in Toxoplasma gondii
Source: Parasit Vectors. 2024 Mar 18;17:142. doi: 10.1186/s13071-024-06135-6 (PMC10949797; doi:10.1186/s13071-024-06135-6)
Supplement: Supplementary file 1 — Additional file 1: Figure S1. Diagnostic PCR of the PPKL-AID line. The integration of the AID fragment at the endogenous locus was assayed by PCR, which showed the absence of the fragment in the parental line (1) but the presence of the fragment in the AID fusion line (2). Figure S2. Organelle stability assay in the PPKL-AID parasites grown in auxin. (A) Parasites were grown in the presence or absence of auxin for 24 h, followed by IFA using organelle protein markers ACP for the apicoplast, ROP5 for the rhoptries, IPT for the endoplasmic reticulum, Cen1 for the centriole, MIC2 for the micronemes, and GRA7 for the dense granule. The protein markers GAP45 and IMC1 were used to label the parasite outline for observing the morphology. Three independent experiments were performed, with similar outcomes. (B) Extracted cytoskeleton in the parasites. Parasites were grown in the absence or presence of auxin, followed by extraction of the cytoskeleton by deoxycholate treatment and tubulin detection. Scale bars = 2 μm. Figure S3. Potential interacting proteins identified by TurboID. (A–C) IFA and western blot detection of the TurboID-PPKL fusion line. (A) The parasites were grown for 24 h and processed for IFA using antibodies against Ty and GAP45 for detection of the fusion protein. (B–C) Detection of biotinylated proteins in the TurboID-PPKL line grown in the absence (−) or presence (+) of biotin (500 μM) for 1 h. Fluorescent streptavidin reagents were used to detect biotinylated proteins in the parasite line. Actin served as the control for western blot. (D) Analysis of differential candidates identified in the proximity proteome of PPKL. The volcano plot depicts fold changes in the proteome by comparing the hits in the TurboID with those in the parental line. The P-values and fold changes were analyzed and plotted by log10 and log2, respectively. The heat map is shown for the log10 of P-values. The hits with P<0.05 were labeled with numbers listed by sequential orders in t [file 13071_2024_6135_MOESM1_ESM.docx]

**December 26, 2023**

**The Plant-Like Protein Phosphatase PPKL Regulates Parasite Replication and Morphology in *Toxoplasma gondii***

Xi-Ting Wu^1,*^, Xu-Wen Gao^1,*^, Qiang-Qiang Wang^1,*^, Kai He^1^, Muhammad Saqib Bilal^2^, Hui Dong^1^, Yi-Dan Tang^1^, Hui-Yong Ding^1^, Yue-Bao Li^1^, Xiao-Yan Tang^1^ and Shaojun Long^1,#^

^1^National Animal Protozoa Laboratory and School of Veterinary Medicine, China Agricultural University, Beijing 100193, China

^2^The Key Laboratory of Plant Immunity, Nanjing Agricultural University, Nanjing 210095, China

*These authors contributed equally to the work

^#^Correspondence: Shaojun Long, Email: [LongS2018@163.com](mailto:LongS2018@163.com)

Running title: PPKL regulates parasite replication and morphology in *T. gondii*

**This file includes Figure S1-3 and their Legend, Table S1-3 and legend for Table S4**


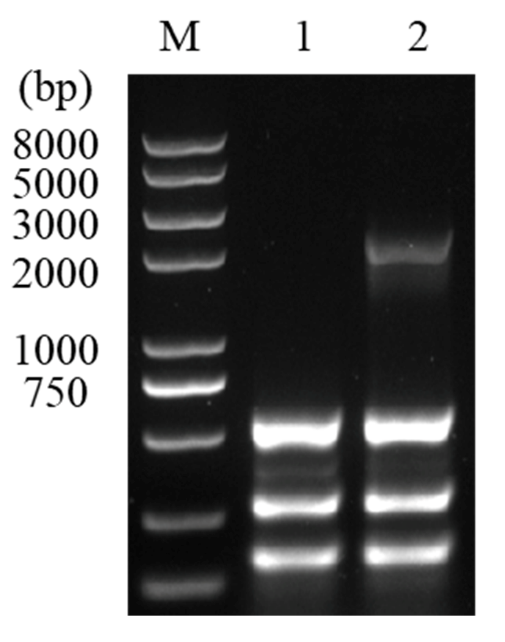


**Figure S1. Diagnostic PCR of the PPKL-AID line.** The integration of the AID fragment at the endogenous locus was assayed by PCR, which showed the absence of the fragment in the parental line (1), but the presence of the fragment in the AID fusion line (2).


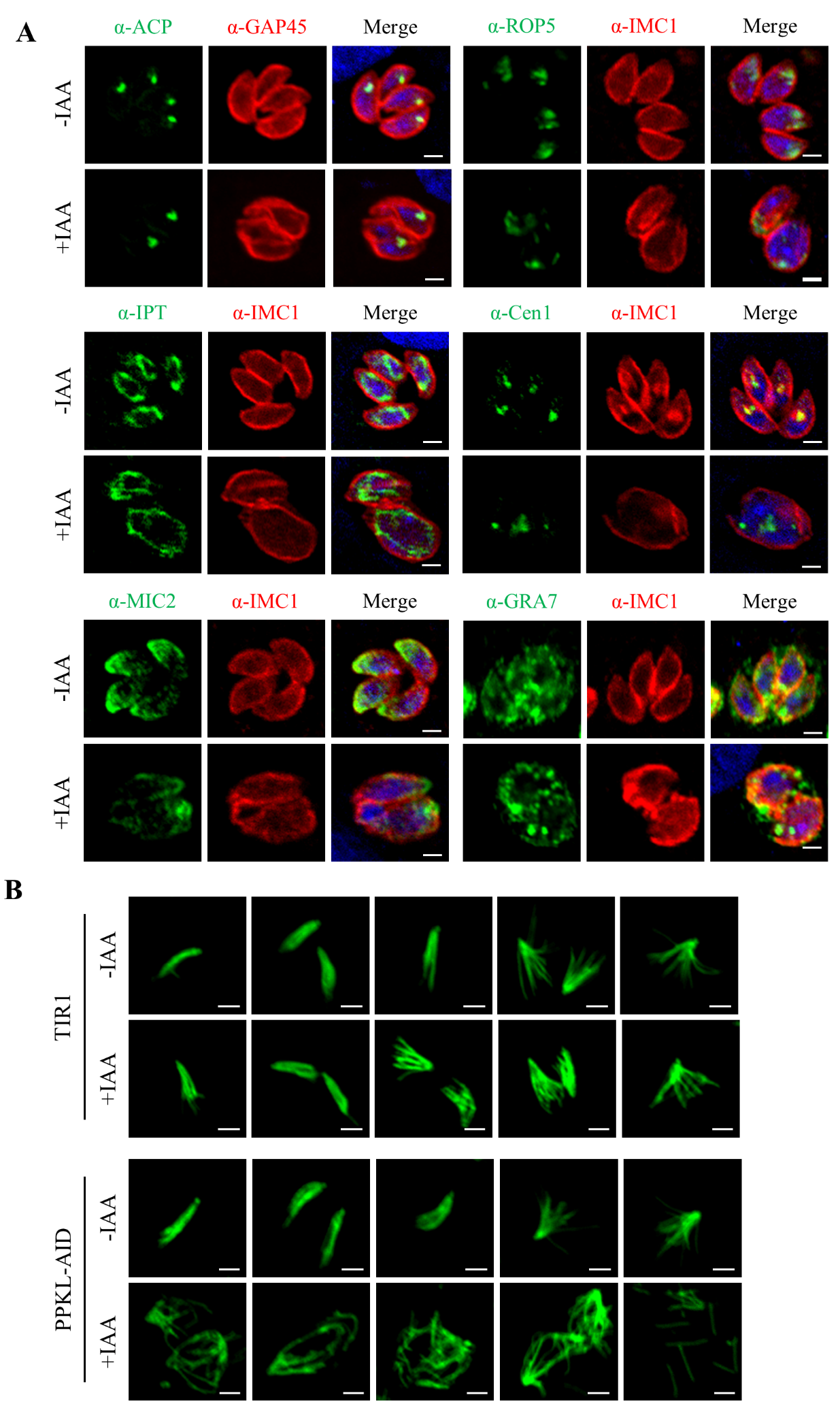


**Figure S2. Organelle stability assay in the PPKL-AID parasites grown in auxin.** (A) Parasites were grown in the presence or absence of auxin for 24 hours, followed by IFA analysis using organelle protein markers, such as ACP for the apicoplast, ROP5 for the rhoptries, IPT for the endoplasmic reticulum, Cen1 for the centriole, MIC2 for the micronemes, GRA7 for the dense granule. The protein markers of GAP45 and IMC1 were used to label the parasite outline for observing the morphology. Three independent experiments were performed with similar outcomes. (B) Extracted cytoskeleton in the parasites. Parasites were grown in the absence or presence of auxin, followed by extraction of the cytoskeleton by deoxycholate treatment and tubulin detection. Scale bars = 2 μm.


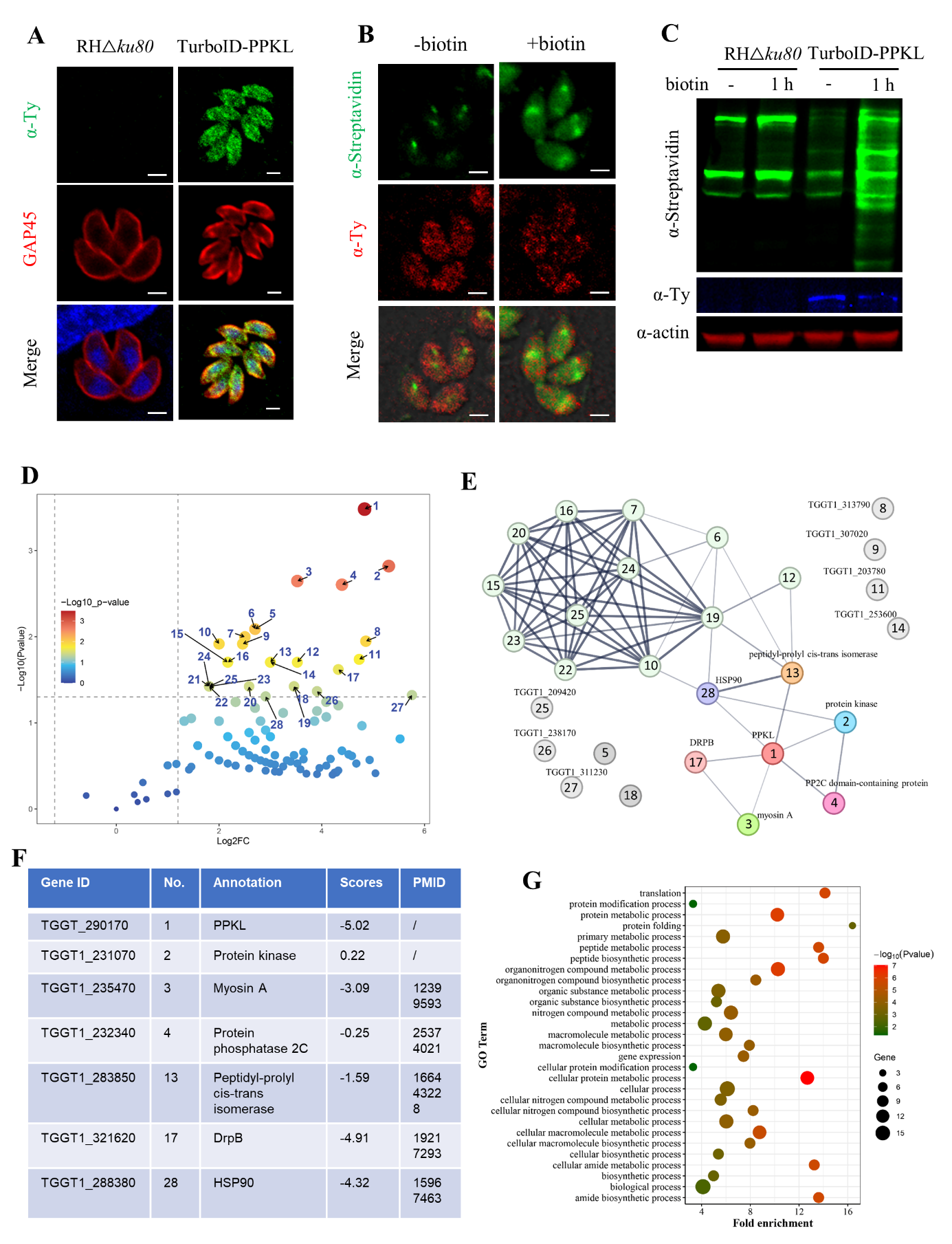


**Figure S3. Potential interacting proteins identified by TurboID.** (A-C) IFA and Western blot detection of the TurboID-PPKL fusion line. (A) The parasites were grown for 24 hours and processed for IFA analysis using antibodies against Ty and GAP45 for detection of the fusion protein. (B-C) Detection of biotinylated proteins in the TurboID-PPKL line grown in absence (-) or presence (+) of biotin (500 μM) for 1 hour. The fluorescent streptavidin reagents were used to detect the biotinylated proteins in the parasite line. The actin served as the control for Western blot. (D) Analysis of differential candidates identified in proximity proteome of PPKL. The volcano plot depicts fold changes in the proteome by comparing the hits in the TurboID to the parental line. The *p* values and fold changes were analyzed and plotted by log10 and log2, respectively. The heat map was shown for the Log10 of *p* values. The hits with *p*<0.05 were labelled with numbers listed by sequential orders in the table (Table S4). (E-F) The interaction of differential protein candidates retrieved from (D) was predicted using the STRING database, and the sequential order numbers were listed in the interacting network (E). The candidates in the core interactome of PPKL were listed with information on CRISPR fitness scores (34), the sequential order, and known information published. (G) Gene Ontology analysis of the 28 proteins with p<0.05. The hits were identified by statistic analysis in (D), and uploaded for GO analysis in ToxoDB.

**Table S4.** Statistical analysis of mass-spectrometry datasets identified in the purified samples from the parental line and the TurboID-PPKL line using streptavidin beads. The foldchanges and *p* values were calculated by comparing the peptide numbers of the TurboID-PPKL (PPKL_1/2) to those of the parental line (RH_1/2). Note that the peptide number with zero in the RH were considered as 1 for the analysis. The resultant results were used to plot the figure S3D, and the hits with p<0.05 were used for protein interaction analysis by STRING (Fig S3E), and Gene ontology analysis in ToxoDB (Fig S3G).

**Table S1. Lines used in this study.**

| **No.** | **Name** | **Genotype** | **Aim** |
| --- | --- | --- | --- |
| 1 | PPKL-AID | RHΔ*ku80*Δ*hxgprt*; TUB1:TIR1-3FLAG, SAG1:CAT; PPKL-AID-6TY, DHFR-TS:DHFR | Conditional knockdown |
| 2 | PPKL-COMP | RHΔ*ku80*Δ*hxgprt*; TUB1:TIR1-3FLAG, SAG1:CAT; PPKL-AID-6TY, DHFR-TS:DHFR; TgPPKL: HA-TgPPKLwt, DHFR-TS:HXGPRT | Complementation |
| 3 | ME49Δ*ku80*/TIR1/PPKL-AID | ME49Δ*ku80*; TUB1:TIR1-3FLAG, SAG1:CAT; PPKL-AID-6TY, DHFR-TS:DHFR | Conditional knockdown |
| 4 | TurboID-PPKL | RHΔ*ku80*Δ*hxgprt*; Ty-TurboID-PPKL, DHFR-TS:DHFR | Biotinylation |

**Table S2. Plasmids used in this study.**

| **No.** | **Plasmids Name** | **Genotype** | **Application** |
| --- | --- | --- | --- |
| 1 | pCas9-PPKL sgRNA 3’ | SAG1:Cas9, U6: EPS15 sgRNA 3’ | C-terminal Tagging |
| 2 | pCas9-PPKL sgRNA 5’ | SAG1:Cas9, U6: EPS15 sgRNA 5’ | N-terminal Tagging |
| 3 | pLinker-AID-6Ty-DHFR-LoxP | Linker-AID-6Ty, LoxP-DHFR-TS:DHFR-LoxP | C-terminal Tagging |
| 4 | pNL-Ty-TurboID-DHFR-LoxP | DHFR-TS:DHFR, RNG2:Ty-TurboID | N-terminal Tagging |
| 5 | pNL-HA-AID-HXGPRT-LoxP | DHFR-TS:HXGPRT, RNG2:HA-AID | N-terminal Tagging |
| 6 | pHA-TgPPKLwt-HXGPRT | DHFR-TS:HXGPRT, TgK13:HA-TgPPKLwt | Complementation |

**Table S3. Primers used in this study**

|  | **Templates** | **Primer pairs and sequence (5’→3’)** | **Fragments** |
| --- | --- | --- | --- |
| Construction of pCas9 plasmid | pCas9 | F1: GAAACAGCAGATTCGCCTG | F1 |
|  |  | R1: AACTTGACATCCCCATTTACCAG |  |
|  | pCas9 | F2: TCGGTCCTCCGATCGTTG | F2 |
|  |  | R2: CAGGCGAATCTGCTGTTTC |  |
|  | pCas9 | F3: TAAATGGGGATGTCAAGTTCGAAGGAGAGAAAAGTTTGCGTTTTAGAGCTAGAAATAGC | F3(PPKL 3’sgRNA) |
|  |  | R3: CAACGATCGGAGGACCGA |  |
|  | pCas9 | F4: TAAATGGGGATGTCAAGTTGCCGGCGGAGTGGAGAGAAGGTTTTAGAGCTAGAAATAGC | F3(PPKL 5’sgRNA) |
|  |  | R3: CAACGATCGGAGGACCGA |  |
| C-terminal Tagging | pLinker-AID-6Ty-DHFR-LoxP | PPKL-L: GCTGCACCGCGCATGCGAGAGACAGACTTTGGTGCGGGGGGGGCTAGCAAGGGCTCGGG | homologous fragment |
|  |  | PPKL-T: GAAGATTGCGATCGAACGCAAGATGCGTTCCGCATTCCAGCACGAATTGGAGCTCCACC |  |
| N-terminal Tagging | pNL-Ty-TurboID-DHFR-LoxP | PPKL-M: TCTCTCTCGCCCTCTGTGGTCGTCCTGCGAGGCCTCGCTTCGTAAAACGACGGCCAGT | homologous fragment |
|  |  | PPKL-NL: GAAGGTAGACCGGGCAGACCATTCTCATGCAGCTGGTTCATTTTGTCCGATGCCGAGCC |  |
| Detection of PPKL-AID strain | *T.gondii* DNA | F5: TGCGCGAAGACCCTTTCGAT |  |
|  |  | R5: CGGCCGACAGGACGCTACTG |  |
| Detection of TurboID-PPKL strain | *T.gondii* DNA | F6: TCCGAATTCTTTGGGTGAC |  |
|  |  | R6: ACTCCCAGAAACTCGGAC |  |
| Construction of complement plasmid | pNL-HA-AID-HXGPRT-LoxP | F7: TTTGGTGCGGGGGGGTGAGATATCAAGGGATCGGGC | F1 |
|  |  | R7: CACGTCATACGGGTACATTTTGTCGCTAGCCCTAGG |  |
|  | *T.gondii* cDNA | F8: ATGTACCCGTATGACGTGCCGGATTACGCGGGCAACCAGCTGCATGAGAAT | F2 |
|  |  | R8: TCACCCCCCCGCACCAAAGTCTG |  |
| Detection of complement plasmid | complement plasmid | F9: CGTTCCATGTAAGGCCAG |  |
|  |  | R9: TTTGTCCGATGCCGAGCC |  |
